# A Cysteine-Reactive Small Photo-Crosslinker Possessing Caged-Fluorescence Properties: Binding-Site Determination of a Combinatorially-Selected Peptide by Fluorescence Imaging/Tandem Mass Spectrometry

**DOI:** 10.3390/ijms19113682

**Published:** 2018-11-21

**Authors:** Kazuki Yatabe, Masaru Hisada, Yudai Tabuchi, Masumi Taki

**Affiliations:** Department of Engineering Science, Bioscience and Technology Program, The Graduate School of Informatics and Engineering, The University of Electro-Communications (UEC), 1-5-1 Chofugaoka, Chofu, Tokyo 182-8585, Japan; y1733131@edu.cc.uec.ac.jp (K.Y.); h1633078@gmail.com (M.H.); t1833099@edu.cc.uec.ac.jp (Y.T.)

**Keywords:** binding-site determination, combinatorially-selected peptide, caged fluorophore, pharmacophore, photoaffinity labeling (PAL), bioisostere, cysteine-reactive crosslinker, fluorescence imaging, tandem MS analysis, covalent binder

## Abstract

To determine the binding-site of a combinatorially-selected peptide possessing a fluoroprobe, a novel cysteine reactive small photo-crosslinker that can be excited by a conventional long-wavelength ultraviolet handlamp (365 nm) was synthesized via Suzuki coupling with three steps. The crosslinker is rationally designed, not only as a bioisostere of the fluoroprobe, but as a caged-fluorophore, and the photo-crosslinked target protein became fluorescent with a large Stokes-shift. By introducing the crosslinker to a designated sulfhydryl (SH) group of a combinatorially-selected peptide, the protein-binding site of the targeted peptide was deduced by sodium dodecyl sulfate-polyacrylamide gel electrophoresis (SDS-PAGE)/fluorescence imaging followed by matrix-assisted laser desorption ionization-time of flight tandem mass spectrometry (MALDI-TOF-MS/MS) analysis.

## 1. Introduction

Recently, targeted peptides in which solvatochromic fluoroprobes are position-specifically introduced [1,2,3], are attracting attention for the rapid and readily interpretable detection of their targets, such as proteins [4,5,6,7,8,9], carbohydrates [10], or biomolecular interactions [9,11]. To rationalize the targeted bindings and to determine the binding sites, in silico docking simulations between the targeted peptides and the proteins are often performed [5,6,12]. The docking between peptide/protein is believed to be more challenging than that between small-molecule/protein, mainly because conformation of the peptide is too flexible to predict the most appropriate binding geometry to the target protein [13]. Experimentally, the binding-site determination is dominantly performed by NMR (Nuclear magnetic resonance) spectroscopy [14] or X-ray crystallography. While the privilege of the X-ray analysis is that the whole structure can be obtained at once, the advantage of the NMR method over X-ray analysis is that it can be used with proteins in the solution state; for the latter, we have to crystallize the peptide/protein complex, which is usually difficult and sometimes impossible. To perform the protein-based NMR analysis in the solution state, the target protein should be labeled with expensive stable isotopes (e.g., ^15^N, ^13^C, or ^2^H) during the translation process. Moreover, high-field NMR systems (above 600 MHz proton frequency) are essential for the precise NMR analysis at the atomic level. As such, there is demand for more conventional bench-side methodologies for binding-site determination.

**Figure 1:**
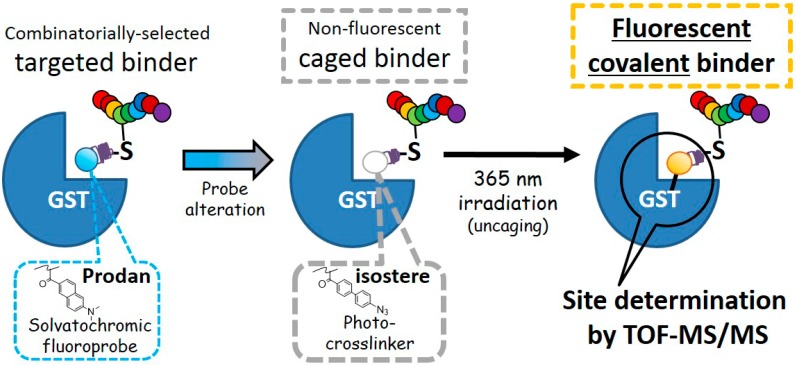
Determining the binding-site of a combinatorially-selected peptide using a rationally designed photo-crosslinker, which is a bioisostere of the solvatochromic fluoroprobe present in the parent peptide. Irradiation with UV light simultaneously crosslinks the fluorophore to the protein binding site and uncages the fluorescence property by forming an intramolecular charge transfer (ICT) structure. This facilitates the rapid deduction of the binding site of the peptide using SDS-PAGE with fluorescence imaging followed by tandem MS analysis. The dotted line stands for the fluorescent color of each probe.

Mass spectrometry-based structural analysis in combination with crosslinking [15,16,17,18] between the targeted peptide and the protein is an alternative methodology for binding-site determination without using X-ray or labeled-NMR techniques. For example, a photo-crosslinking amino acid that is structurally similar to the corresponding natural amino acid (Figure S1), is incorporated at a specific position of the targeted peptide [18,19,20] to obtain a bioisostere [21]. Then, the bioisostere peptide/target protein complex is covalently conjugated under irradiation, and the crosslinked protein is digested by trypsin. The resulting fragments of the targeted-peptide/protein conjugate are identified by tandem MS analysis. Before the digestion followed by the MS analysis, confirmation of the successful crosslinking by SDS-PAGE would be favorable. Sometimes, as the crosslinking efficiency is not so high, and/or the gel mobility of the crosslinked product is not distinguishable from that of the unreacted one, the crosslinked protein is often detected by in-gel fluorescent imaging [12,22,23,24]. In many cases, such a fluorescent “covalent” binder contains three components: a fluoroprobe, a linker, and a target-recognizing element (i.e., pharmacophore). However, attaching such fluoroprobes, especially big ones, often perturbs the target-binding properties [12], including target specificity. To avoid this, one can conjugate a small reactive tag (e.g., an alkyne) to the binder in advance, and the fluoroprobe is successively attached to the tag after photo-crosslinking [25,26]. When not using this strategy, more preferably, the fluorophore would not only work as a crosslinker [23,24] but also be one part of the pharmacophore [27]. In this context, here, we synthesized a cysteine-reactive small photo-crosslinker [19,22] possessing the caged-fluorescence property of azide [27,28]; the novel crosslinker was designed as an analog of a solvatochromic fluoroprobe (i.e., Prodan; also see Figure S1) which is a part of a glutathione-S-transferase (GST)-specific targeted peptide [5]. As represented in Figure 1, in order to determine the binding site of the combinatorially-selected targeted peptide obtained via extended T7-phage display technology (i.e., 10BASE_d_-T [5]), Prodan in the binder was altered to the analogous crosslinker as a bioisostere, and its targeted covalent binding toward GST was confirmed by SDS-PAGE, followed by in-gel fluorescent imaging. By using the trypsinized fragment of the crosslinked fluorescent GST, the binding site was deduced by tandem MS analysis.

## 2. Results and Discussion

### 2.1. Synthesis of Cysteine-Reactive Small Photo-Crosslinkers and their Attachment to the Combinatorially-Selected Peptide

**Figure 2:**
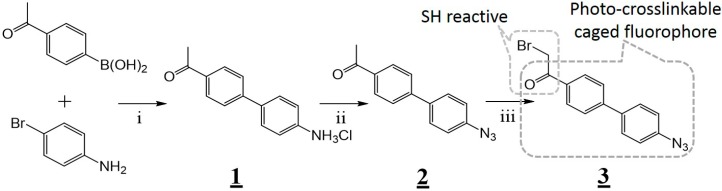
Overall scheme for the synthesis of the cysteine-reactive small photo-crosslinker **3**. Reagents and conditions: (i) K_3_PO_4_, XPhos Pd G2, 1,4-dioxane/H_2_O, reflux, 2 hours; 1.7 M HCl (dichloromethane/ethyl acetate), room temperature, 24 hours, 77%; (ii) NaNO_2_, NaN_3_, 3.4 M aqueous HCl, 0 °C, 3 hours, 84%; (iii) *N*-Bromosuccinimide (NBS), TsOH monohydrate, MeCN, 35 °C, 24 hours, 33%.

To obtain the photo-crosslinkable caged fluorophore in the least number of steps, Suzuki coupling between 4-acetylphenylboronic acid and 4-bromoaniline was performed, and the resulting twisted intramolecular charge transfer (TICT [29])-based fluorophore was azidated for the caging (Figure 2). As we expected, the azidated caged-fluorophore (**2**) absorbed 300–400 nm ultraviolet light (Figure S2B). It was gradually uncaged to became fluorescent (Figure S2C) with a large Stokes-shift (168 nm; Figure S2D) by photo-reduction of the azide to amine [30] under exposure to UV light using a handlamp. The aryl-azide structure is also frequently utilized for photoaffinity labeling (PAL) [31]; therefore, we expected that the crosslinking would simultaneously occur along with the uncaging [27], if the azidated caged-fluorophore was surrounded closely by target protein. Next, the alpha-position of the ketone group of the caged fluorophore was brominated to add reactivity towards cysteine group. Then, the resulting photo-crosslinker was conjugated with a designated cysteine (underlined) of a combinatorially-selected peptide whose sequence is NTVSCHGF [5], to obtain the caged binder for GST (Figure S6). At this stage, the conjugated caged fluorophore would work as a bioisostere of Prodan, which is known to be a part of the pharmacophore for GST-binding [5].

### 2.2. Targeted Covalent Conjugation of Caged Binder with GST

**Figure 3:**
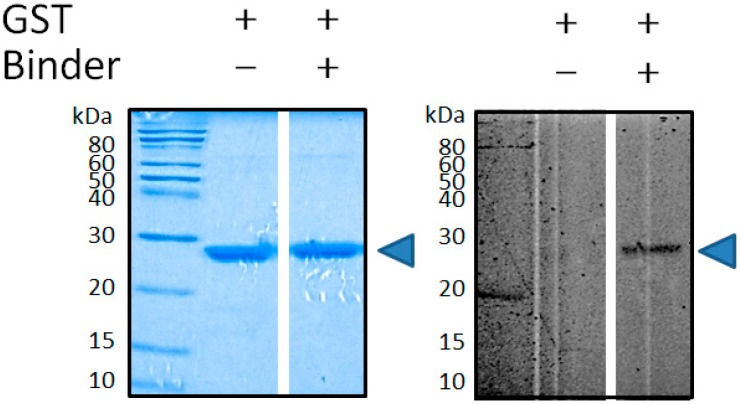
Specific conjugation between glutathione-S-transferase (GST) and the caged binder, confirmed by 15% SDS-PAGE/fluorescence imaging. GST (blue arrow) was visualized by CBB staining (left panel), and the binder-conjugated GST was visualized by fluorescence in the same gel (right panel). For the fluorescence imaging, the excitation wavelength was 405 nm, and a band-pass filter (605 nm) was used for the detection. Plus (+) and minus (-) stand for presence and absence of the suggested molecules (i.e., GST or the binder), respectively.

For the rapid confirmation of its successful crosslinking against the target protein, the caged binder and GST were incubated, irradiated with long-wavelength ultraviolet light (365 nm), and examined by SDS-PAGE. After exposure to the light, the characteristic yellow fluorescence was immediately observed, regardless of the presence or absence of the target protein. This suggests that the expected uncaging of the caged binder occurred successfully within a short period: First, N_2_ elimination from the aryl-azide moiety was conducted to form the corresponding nitrene intermediate [32] (Figure S2A). Then, addition or an active-hydrogen insertion reaction between the intermediate and the target protein and/or solvent seemed to occur, to form the expected TICT-fluorescence structure; most of the caged binders tended to react with the surrounding solvent molecule. Nevertheless, its appropriate crosslinking with GST was confirmed by SDS-PAGE, followed by fluorescence imaging/Coomassie brilliant blue (CBB) staining. After the electrophoresis, a yellow fluorescent band was seen at the appropriate molecular weight (ca. 29 kDa) of the binder-conjugated GST (Figure 3). Judging from time-conversion experiment on the basis of SDS-PAGE densitometric analysis of the fluorescent band, the crosslinking reaction ended within 30 seconds (data not shown).

**Figure 4:**
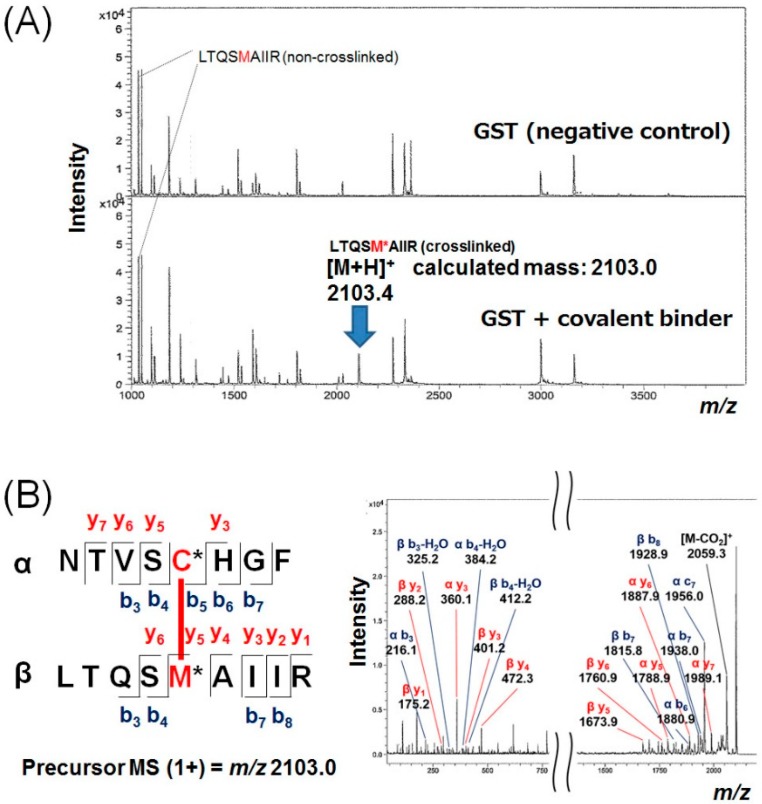
(**A**) Identification of trypsinized peptide fragments derived from the covalent-binder-conjugated GST by MALDI-TOF-MS analysis (lower panel). As a negative control, pristine GST was also trypsinized, and the resulting fragments were also analyzed under the same conditions (upper panel). A newly appeared fragment in the presence of the covalent binder is highlighted by a blue arrow. (**B**) MS/MS spectra of the newly appeared fragment. All the detected fragments were consistent with theoretical *m*/*z* values of the represented structure; b- and y-ions are highlighted by using blue and red colors, respectively. The peptide fragment of LTQSMAIIR was derived from a constituent of the glutathione binding pocket of GST protein; judging from the peak intensity of the remaining non-crosslinked fragment (lower panel in A), the crosslinking reaction yield was estimated to be a few percent. M* and C* mean conjugated methionine and cysteine, respectively; the conjugation is highlighted by a thick red line.

To determine the site/position specificity of the crosslinking, the fluorescent GST band in the gel was excised and digested with trypsin. The resulting peptide fragments were analyzed by MALDI-TOF-MS/MS. An intense peak possessing a mass divided by charge number (*m*/*z*) of 2103 was newly detected after the conjugation, and it was identified as the GST-crosslinked peptide fragment by MS (Figure 4A) and tandem mass spectroscopy (Figure 4B). The crosslinking site was determined to be the glutathione binding pocket; the crosslinked amino acid was a methionine that was located at the 69th position from the N-terminus. Judging from the mass number, the detailed chemical structure of the crosslinked product was also tentatively deduced, and the possible structures were depicted in Figure S7. The covalent bonding was formed on the methyl (CH_3_) group, which is connected next to the sulfur atom of the methionine side-chain.

### 2.3. Molecular Docking Simulation for Rationalizing Cross-Linking Specificity

**Figure 5:**
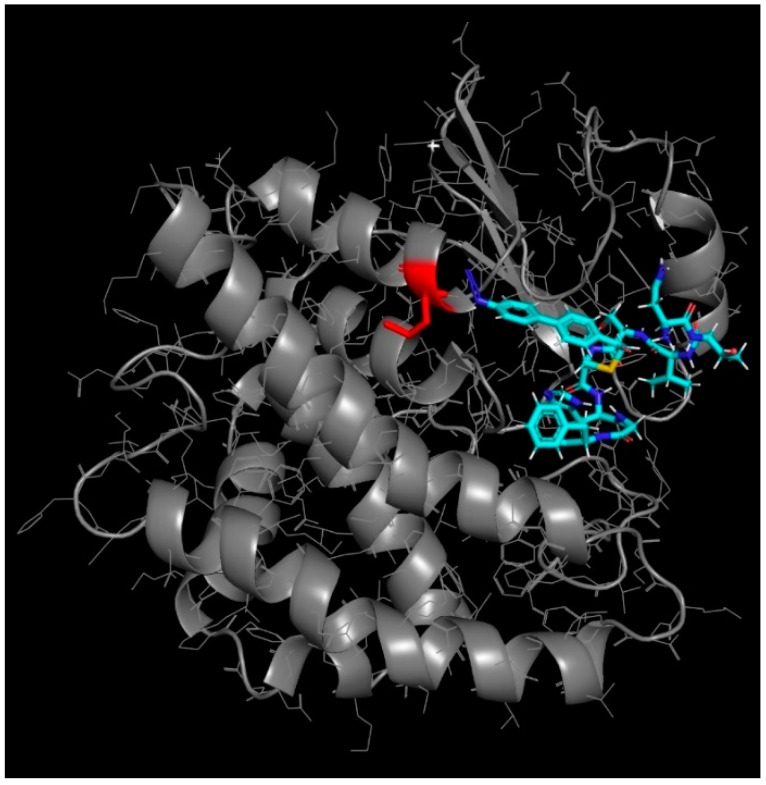
Molecular docking simulation of the caged binder (shown as a stick; C, N, O and S atoms are highlighted by cyan, blue, red and yellow colors, respectively) to GST (PDB ID: 1UA5) using the sievgene of myPresto; the best docking model with a lowest binding energy of −12.0 kcal/mol was presented. The azido group (i.e., N_3_) in the caged fluorophore and conjugated methionine in GST were colored in blue (double-lined) and red, respectively. GST was shown as a cartoon with side chains as a line description. Including this lowest model, 23 independent models out of the 30 separate poses resulted that the azido group was also closely located to the methionine.

Finally, the crosslinking of the caged binder was rationalized by a protein–ligand docking simulation using sievgene [33] of myPresto [34]. Thirty separate poses resulted in docking to the glutathione binding pocket of GST with free energy in the range −12.0 to −8.18 kcal/mol. As shown in Figure 5, the docking model of the lowest energy suggested that the photo-crosslinkable caged fluorophore was buried deep inside the hydrophobic region of the glutathione-binding pocket and located very close to the conjugated methionine of the 69th position, which probably resulted in site- and position-specific crosslinking. The geometry of the whole peptide (NTVSC*HGF; C*: caged fluorophore) was in good agreement with the experimentally-supported docking simulation of Prodan-conjugated NTVSC*HGF [5], which suggests that the caged fluorophore successfully worked as a bioisostere of Prodan (i.e., a part of the pharmacophore against GST).

## 3. Materials and Methods

### 3.1. General

All of the detailed experimental procedures, including the chemical synthesis of the novel cysteine-reactive small photo-crosslinker possessing caged fluorescence property and its conjugation with the combinatorially-selected peptide to afford the caged GST-binder, as well as the molecular docking simulations, were described in the electronic Supplementary Materials.

### 3.2. Photo-Crosslinking of the Caged Binder and GST

Photo-crosslinking of a recombinant GST (50 μM) with the caged binder (0.50 mM) was carried out with the irradiation of a 365 nm light by using a handheld UV lamp (6 W, UVGL-58, 100 V; Funakoshi, Japan) for 5, 10, or 30 sec, or 1–20 min in a solution containing D-PBS (pH 7.4)/DMSO (*v*/*v*, 4:1, total volume 10 μL) at room temperature. A small portion of the resulting product was then subjected to 15% SDS-PAGE (shown in Figure 3). After the electrophoresis, the crosslinked protein was visualized by in-gel fluorescence imaging using a fluoroimager (FMBIO III-SC01, Hitachi, Japan). More conveniently, it could be also visualized by the naked eye or through a smartphone using the handheld UV lamp as an excitation source. GST was also visualized by a rapid stain CBB kit (Nacalai, Japan), and gel imaging was performed by a conventional gel imager (Gel Doc XRS+; Bio-Rad, Hercules, CA, USA).

### 3.3. In-Gel Trypsinization of Crosslinked GST and Mass Spectrometric Analysis

Photo-crosslinking of GST (0.27 mM) with the caged binder (1.3 mM) was once again carried out under irradiation for 20 min in a solution containing phosphate buffer (20 mM, pH 7.4)/DMSO (*v*/*v*, 4:1, total volume 60 μL) at room temperature. It was separated by 15% SDS-PAGE using a large gel, and the fluorescent band around 29 kDa was excised from the gel. The protein in the gel was reduced with 25 mM dithiothreitol at 65 °C for 10 min, and then alkylated with 55 mM iodoacetamide at room temperature for 60 min in the dark. Digestion was carried out with modified trypsin (Promega, Madison, WI, USA) at 37 °C overnight, and the digested peptide fragments were desalted by using a ZipTipC18 silica resin (Merck Millipore, Burlington, MA, USA) according to the instruction manual. The desalted peptides were spotted on a stainless plate with α-cyano-4-hydroxycinnamic acid as a matrix, and analyzed by MALDI-TOF-MS using an Autoflex Speed (Bruker Daltonics, Billerica, MA, USA) instrument.

## 4. Conclusions

In conclusion, we have altered a non-natural fluoroprobe in a combinatorially-screened targeted peptide into a caged photo-crosslinker as a bioisostere. When irradiated, the altered peptide covalently bound to GST in association with uncaging (i.e., fluorescence recovery). The binding site of the screened peptide was conveniently deduced by SDS-PAGE/fluorescence imaging, followed by MALDI-TOF-MS/MS analysis. Although the binding-site determination can also be performed by a non-photoaffinity-type covalent binder [12], the reactive warhead (e.g., SO_2_F group) may potentially cause unfavorable crosslinkings such as intramolecular cyclization and off-targeted intermolecular side reactions. Therefore, we speculate that by using a photoaffinity-type covalent binder would attain more promising results for precise structural determination.

## Supplementary Materials

Supplementary materials can be found at http://www.mdpi.com/1422-0067/19/11/3682/s1.

## Abbreviations

NMR	Nuclear magnetic resonance
SDS-PAGE	Sodium dodecyl sulfate-polyacrylamide gel electrophoresis
MALDI-TOF-MS/MS	Matrix-assisted laser desorption ionization-time of flight tandem mass spectrometry
Prodan	6-Propionyl-2-dimethylaminonaphthalene
UV	Ultraviolet
DMSO	Dimethyl sulfoxide
D-PBS	Dulbecco’s phosphate-buffered saline
PDB	Protein Data Bank
