# Supplementary material for: A Cysteine-Reactive Small Photo-Crosslinker Possessing Caged-Fluorescence Properties: Binding-Site Determination of a Combinatorially-Selected Peptide by Fluorescence Imaging/Tandem Mass Spectrometry
Source: Int J Mol Sci. 2018 Nov 21;19(11):3682. doi: 10.3390/ijms19113682 (PMC6274937; doi:10.3390/ijms19113682)
Supplement: Supplementary file 1 [file ijms-19-03682-s001.pdf]

International Journal of
Molecular Sciences

MDPI

Communication

Cysteine-Reactive Small Photo-Crosslinker Possessing Caged-Fluorescence Properties: Binding-Site Determination of a Combinatorially-Selected Peptide by Fluorescence Imaging / Tandem Mass Spectrometry

Kazuki Yatabe †, Masaru Hisada †, Yudai Tabuchi, Masumi Taki *

1. Supporting figures

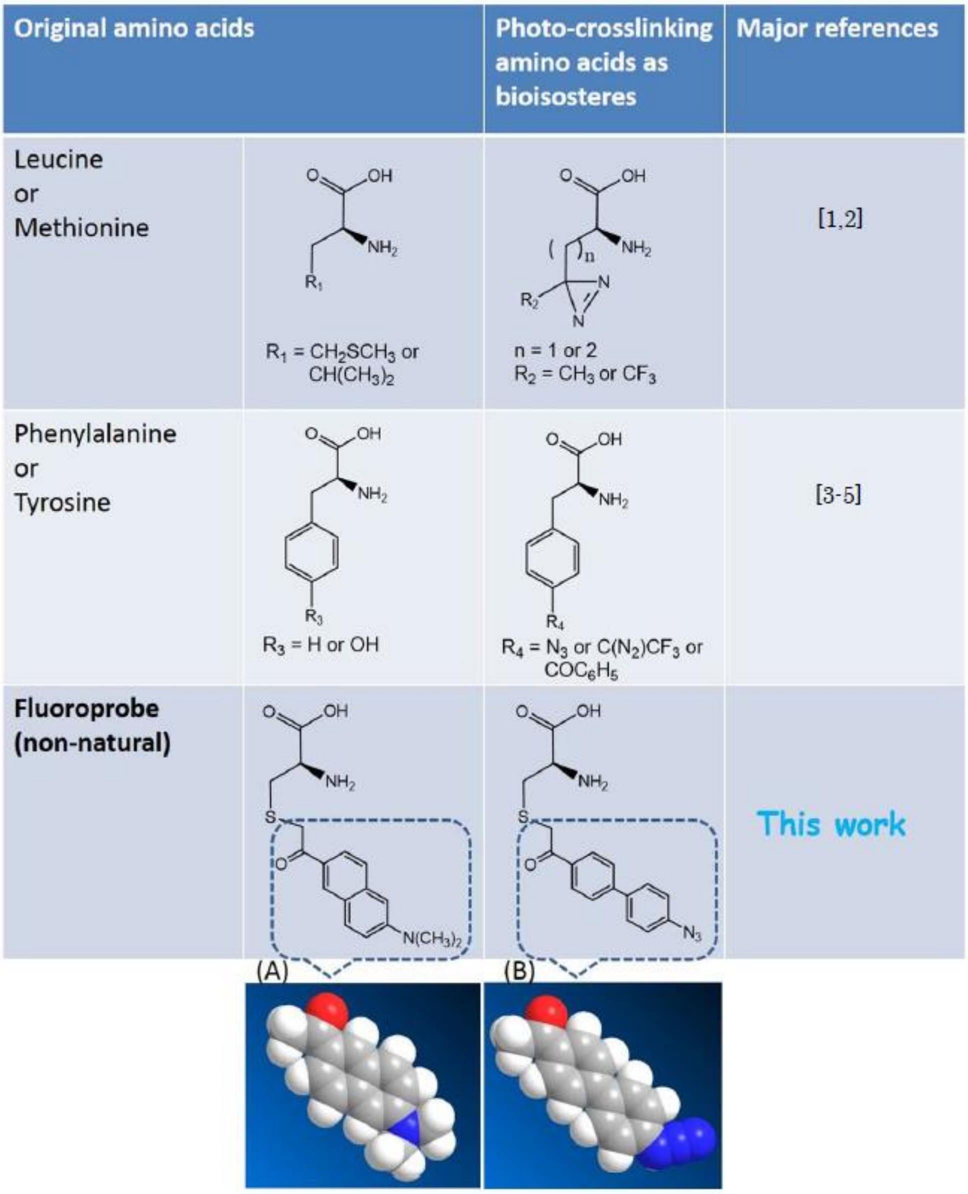

Figure S1. Structure comparison between original amino acids used in target-binding peptides and the corresponding photo-crosslinkers as bioisosteres. The comparison between (A) Prodan

fluoroprobe and (B) its analogous crosslinker possessing caged-fluorescence property is also therein. The natural amino acids shown here are only examples; for the complete description about all of the amino acids and their corresponding photo-crosslinkable bioisosteres, see recent review articles [6,7].

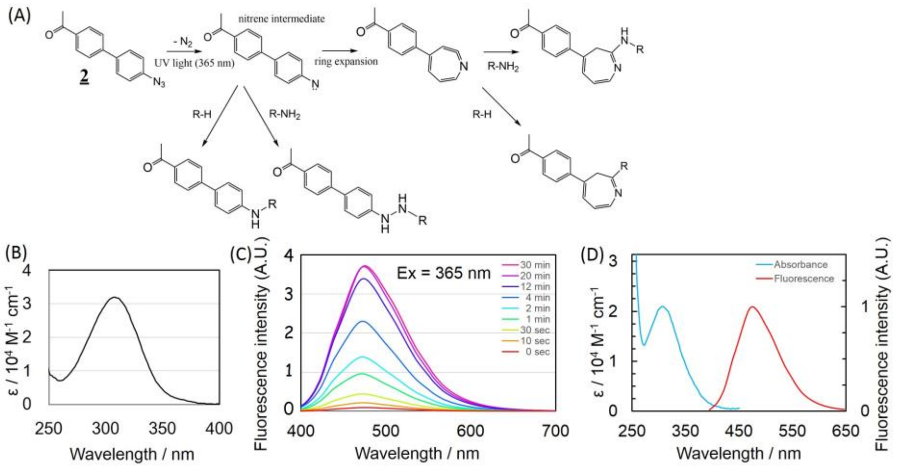

Figure S2. (**A**) Possible products from the photo-crosslinkable caged fluorophore (**2**) [8]. After the irradiation, nitrogen elimination is conducted to form the corresponding nitrene intermediate, and some portion of the intermediate is further transformed to a ring-expanded structure. In either cases, addition or active-hydrogen insertion reaction between the intermediates and the target protein and/or solvent can be occurred. Most probably, the expected twisted intramolecular charge transfer (TICT)-fluorescence structure would be formed without the ring expansion. (**B**) Absorbance spectrum of the caged fluorophore (**2**, 0.10 mM) in DMSO; molar extinction coefficients at 308 nm (i.e., the maximum absorption) and 365 nm were 3.2×10^4 and 1.5×10^3 mol^{-1} L cm^{-1}, respectively. (**C**) Fluorescence spectra of the photo-irradiated products from the caged fluorophore (**2**, 1.0 mM) in DMSO at different irradiation time using a UV handlamp (365 nm); the maximum fluorescence wavelength was 475 nm. (**D**) Absorption (0.10 mM, blue line) and fluorescence (1.0 mM, red line) spectra of the photo-irradiated products from the caged fluorophore (**2**) in DMSO after 30 min exposure to UV light using the handlamp (365 nm). The molar extinction coefficients at 307 nm (i.e., the maximum absorption) and 365 nm were 2.1×10^4 and 5.7×10^3 mol^{-1} L cm^{-1}, respectively, and the Stokes shift was 168 nm.

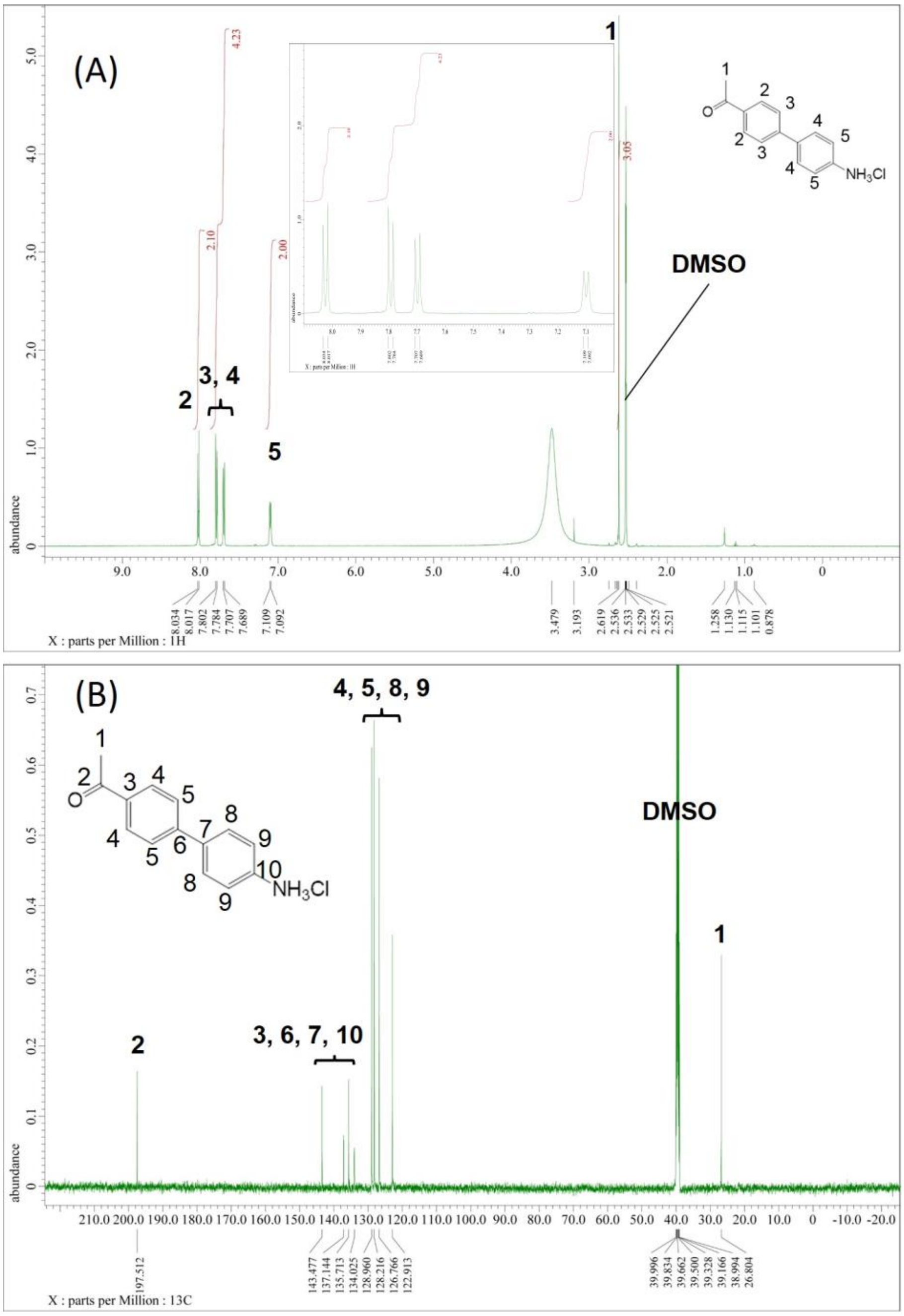

Figure S3. Identification of TICT-fluorescent intermediate (**1**) by (**A**) ^{1}H and (**B**) ^{13}C NMR in DMSO-d_6.

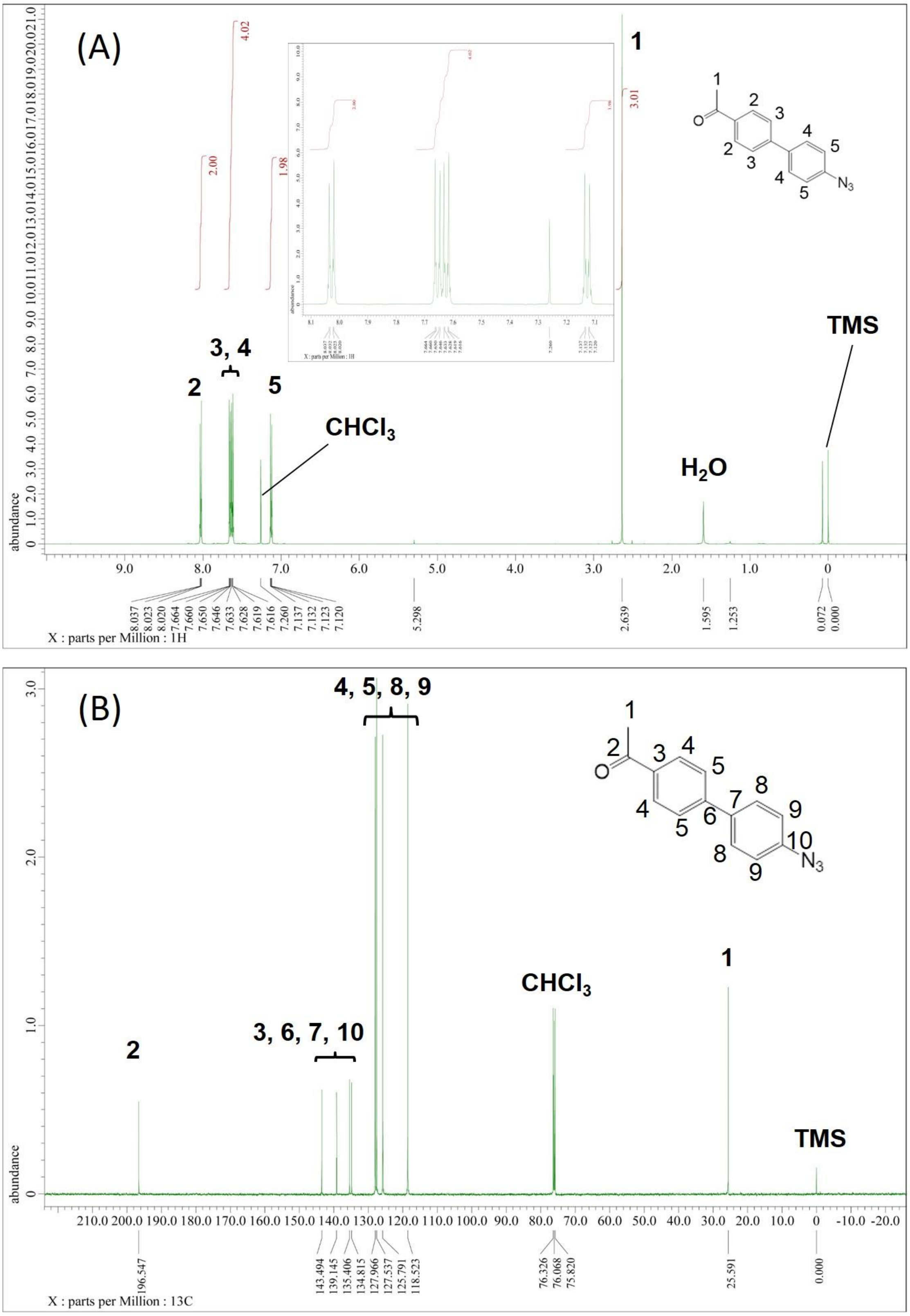

Figure S4. Identification of photo-crosslinkable caged fluorophore (**2**) by (**A**) ^{1}H and **(B**) ^{13}C NMR in $CDCl_3$.

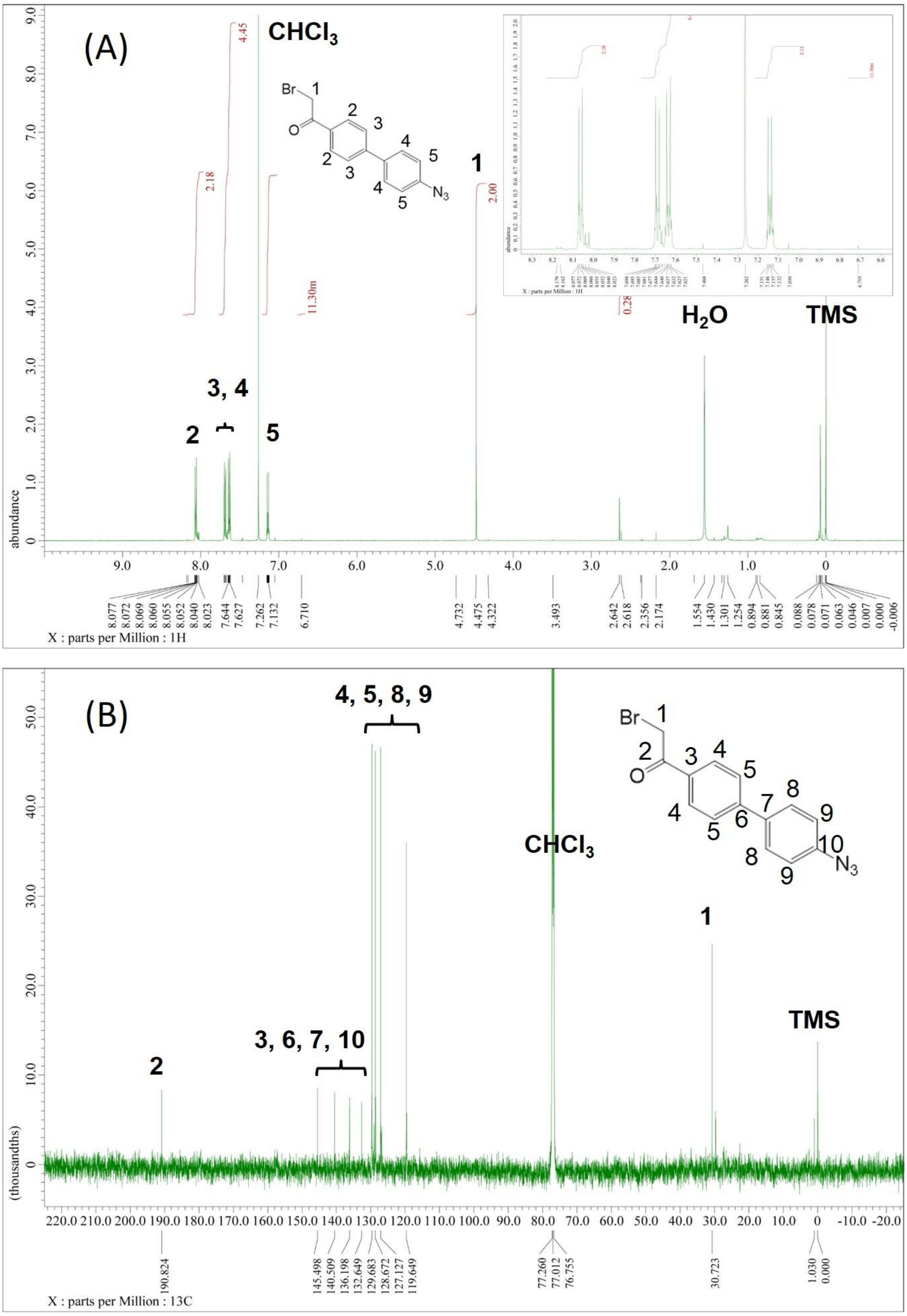

Figure S5. Identification of cysteine-reactive photo-crosslinker (3) by (**A**) ^{1}H and (**B**) ^{13}C NMR in $CDCl_3$.

(A)

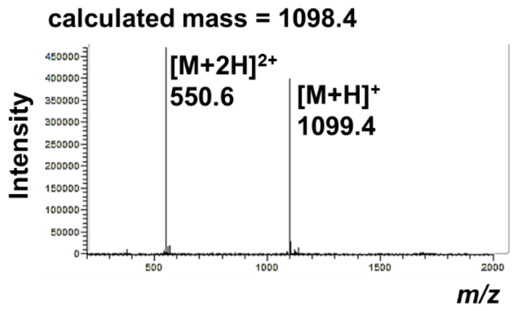

(B)

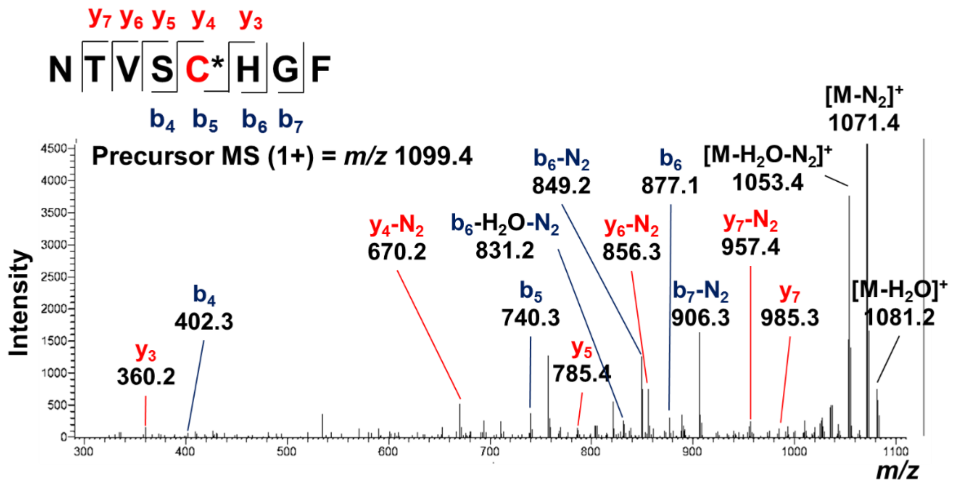

Figure S6. Identification of the purified caged binder (i.e., NTVSC*HGF; C* represents photo-crosslinker-conjugated cysteine) by (**A**) MS and (**B**) tandem mass spectroscopy.

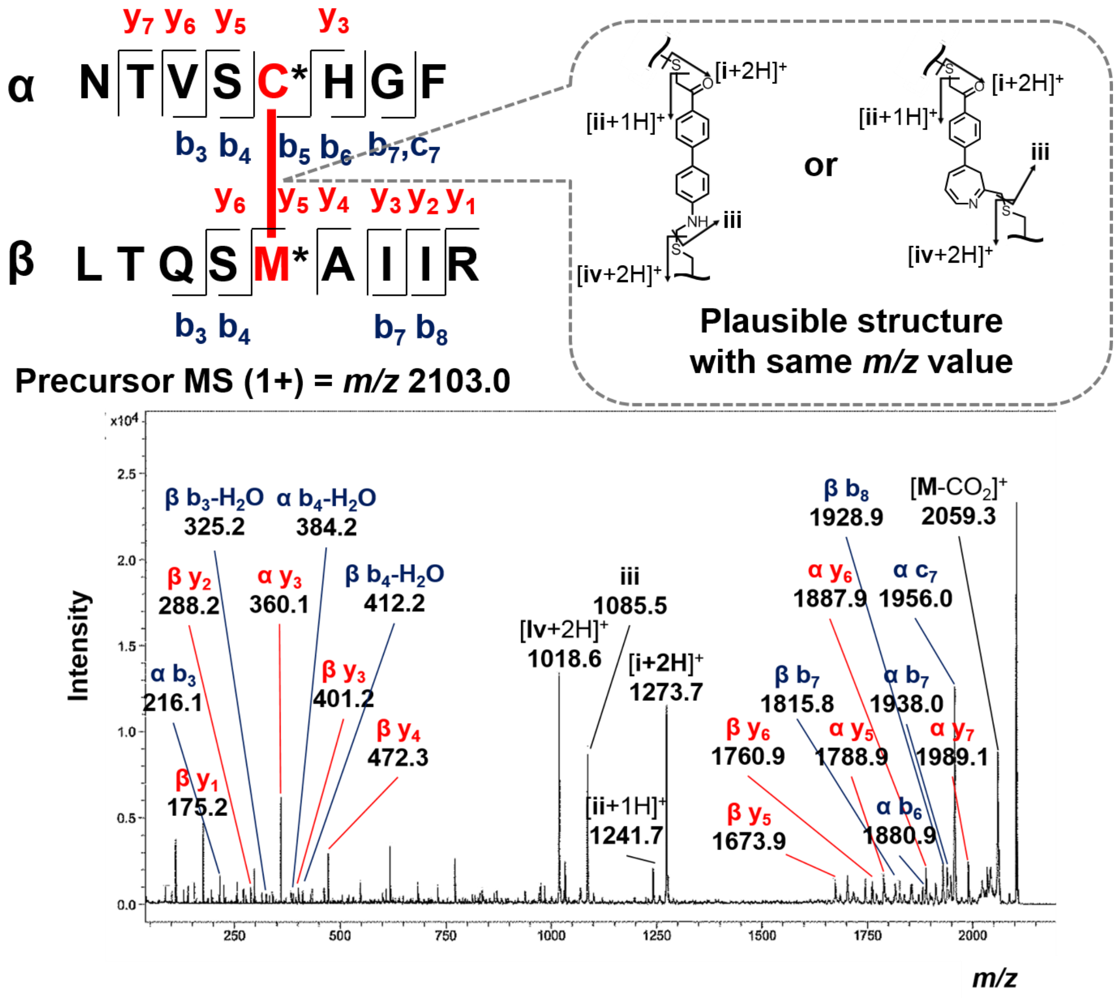

Figure S7. Detailed identification of the crosslinked product between GST and the covalent binder by tandem mass spectroscopy. The plausible chemical structure around the uncaged fluorophore is also depicted.

2. Materials and methods

2.1. General

All of the reagents and solvents are commercially available and used without further purification, except glutathione-S-transferase (GST). GST was prepared according to the reported procedure [9]. Fluorescence measurement was performed on a NanoDrop 3300 (Thermo Scientific) at room temperature; the fluorescence spectra were measured by excitation at 365 ± 10 nm. UV absorbance measurement was performed on a NanoPhotometer P300 (Impren) at room temperature. NMR experiments were performed at 25 °C by using a 500 MHz spectrometer (JNM-ECA500, Jeol Resonance, Japan). All the NMR assignments of the different proton and carbon atoms were deduced by calculations using ChemDraw Ultra (ver. 11.0, CambridgeSoft). The purity of each synthesized fine chemical was determined by integral curves obtained from ^{1}H NMR. Liquid chromatography (LC) analysis was performed on an Agilent 1100 HPLC system (Agilent Technologies) using a 0–100% gradient of acetonitrile containing 0.1% formic acid at a flow rate of 300 μL per minute, equipped with a C18 reverse-phase column (Hypersil GOLD, 2.1 × 100 mm, Thermo Fisher Scientific) connected to a photo diode array (PDA) and/or a LCQ-Fleet ion trap mass spectrometer. Electrospray ionization-time-of-flight-mass spectrometry (ESI-TOF-MS; JMS-T100 AccuTOF, Jeol Resonance, Japan) was performed by dissolving the analyte in methanol and directly injected to the instrument. To confirm

the reproducibility, each experiment (e.g., synthesis, SDS-PAGE / fluorescence imaging, MALDI-TOF-MS analysis) was performed at least twice.

2.2. Synthesis of novel cysteine-reactive small photo-crosslinker possessing caged fluorescence property

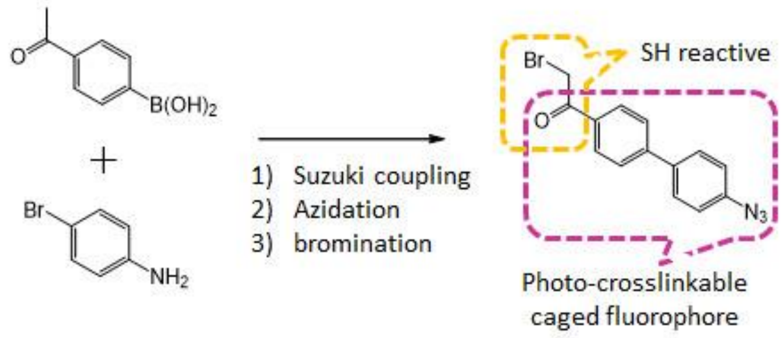

Scheme 1. Overall scheme for synthesis of the cysteine-reactive small photo-crosslinker possessing caged fluorescence property. Each identification was supported by NMR (Figure S3–5) and MS.

2.2.1. Synthesis of TICT-fluorescent intermediate (1)

A 50 mL reaction flask was charged with 4-acetylphenylboronic acid (0.79 g, 4.8 mmol), 4-bromoaniline (0.81 g, 4.0 mmol), K_3PO_4 (1.7 g, 8.0 mmol), and XPhos Pd G2 (63 mg, 80 μmol). Then, 1,4-dioxane / H_2O (v/v, 3:1, 20 mL) were added, and the mixture was refluxed for 2 hours. After the completion of the reaction, 1M NaOH aq. (60 mL) was slowly added to the reaction mixture, and the product was extracted with EtOAc (3 x 20 mL). The combined organic layer was concentrated under reduced pressure. The residue was dissolved in 15 mL of DCM and 4M HCl in EtOAc (10 mL) was added dropwise. The mixture was stirred for 24 hours at room temperature, concentrated under reduced pressure, and purified by recrystallization from MeOH to afford silver-gray crystalline solid (**1**). Identification of **1** was performed by combination of NMR (Figure S3) and ESI-TOF-MS.

1: Yield: 77% (0.76 g); ^{1}H NMR (DMSO-*d*$_6$, 500 MHz) δ 7.98 (d, 2H, *J* =8.6 Hz), 7.76 (d, 2H, *J* = 8.6 Hz), 7.66 (d, 2H, *J* = 8.6 Hz), 7.06 (d, 2H, *J* = 8.6 Hz), 2.58 (s, 3H); ^{13}C NMR (DMSO-*d*$_6$, 125 MHz) δ 197.5, 143.5, 137.1, 135.7, 134.0, 129.0, 128.2, 126.8, 122.9, 26.8; ESI-TOF-MS (*m*/*z*) calcd. for $C_{14}H_{14}NO$: 212.11, found: 212.10 $[M + H]^+$.

2.2.2. Synthesis of photo-crosslinkable caged fluorophore (2)

A 50 mL flask wrapped in aluminium foil was charged with 4M HCl aq. solution (9 mL) of **1** (0.25 g, 1.0 mmol), and incubated for 10 min on ice bath, then $NaNO_2$ (0.11 g, 1.6 mmol) in H_2O (0.5 mL) was added dropwise. After stirring for 30 min, NaN_3 (0.10 g, 1.6 mmol) in H_2O (1 mL) was added dropwise to the reaction mixture, and the solution was stirred on ice bath for additional 2 hours. The reaction mixture was extracted with EtOAc (1 x 20 mL and 1 x 10 mL) and with toluene (1 x 10 mL). The combined organic layer was dried over Na_2SO_4, concentrated under reduced pressure to afford white solid **2**, which was used for the next step without further purification. Identification of **2** was performed by combination of NMR (Figure S4) and UV absorption spectrometry (Figure S2B). Confirmation of the uncaging of **2** was monitored by fluorescence spectrometry after exposure to UV light using a handlamp (Figure S2C).

2: Yield: 84% (0.20 g); ^{1}H NMR ($CDCl_3$, 500 MHz) δ 8.03 (dd, 2H, *J* =3.0 Hz, *J* =4.6 Hz, *J* =1.7 Hz), 7.66 (dd, 2H, *J* =1.7 Hz, *J* =5.2 Hz, *J* =1.7 Hz), 7.62 (dd, 2H, *J* =2.3 Hz, *J* =4.6 Hz, *J* =1.7 Hz), 7.13 (dd, 2H, *J* =2.3 Hz, *J* =4.6 Hz, *J* =1.7 Hz); ^{13}C NMR ($CDCl_3$, 125 MHz) δ 196.6, 143.5, 139.2, 135.4, 134.8, 127.5, 125.8, 118.5, 25.6.

2.2.3. Synthesis of cysteine-reactive photo-crosslinker (3)

A 10 mL flask was charged with a solution of **2** (12 mg, 50 μmol) in MeCN (0.5 mL), then *N*-bromosuccinimide (NBS; 9.5 mg, 53 μmol) and *p*-toluenesulfonic acid (TsOH) monohydrate (9.1 mg, 48 μmol) were successively added. The mixture was reacted for 24 hours at 35 °C. After completion of the reaction, 7% $NaHCO_3$ aq. (13 mL) was added to the reaction mixture. The precipitated solid residue was filtrated and washed with H_2O. The residue was concentrated under reduced pressure, and purified with reverse-phase high performance liquid chromatography (LC-20AD, Shimadzu) equipped with a XTerra Prep MS C18 column (10 x 50 mm, Waters) by using an isocratic elution of 0.1% formic acid / MeOH (30:70, v/v) during 5 min at a flow rate of 4 mL per minute. The eluted fraction was lyophilized completely to afford white solid **3**, which was characterized by NMR (Figure S5).

3: Yield: 33% (4.7 mg, 16 μmol; purity above 90%); ^1H NMR ($CDCl_3$, 500 MHz) δ 8.06 (dd, 2H, *J* =1.7 Hz, *J* =4.6 Hz, *J* =2.3 Hz), 7.70 (dd, 2H, *J* =1.7 Hz, *J* =5.2 Hz, *J* =1.7 Hz), 7.64 (dd, 2H, *J* =2.3 Hz, *J* =4.0 Hz, *J* =2.3 Hz), 7.14 (dd, 2H, *J* =2.3 Hz, *J* =4.6 Hz, *J* =2.3 Hz); ^{13}C NMR ($CDCl_3$, 125 MHz) δ 190.8, 145.5, 140.5, 136.2, 132.7, 129.7, 128.7, 127.1, 119.7, 30.7.

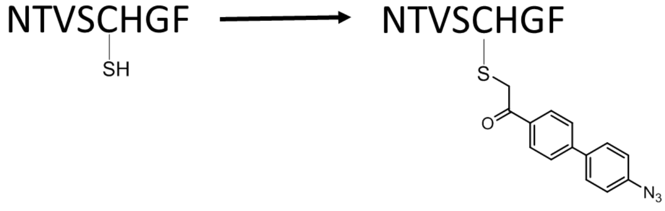

Scheme 2. Synthesis of the caged binder by thioetherification between the photo-crosslinker **3** and a combinatorially-selected peptide [9].

2.3. Synthesis of caged binder

A combinatorially-selected peptide whose sequence is H_2N-NTVSCHGF-OH [9] was synthesized and characterized by GenScript Inc (NJ, USA). SH group in the peptide (2.9 μmol) was reduced beforehand with immobilized TCEP disulfide reducing gel (Thermo Scientific) in phosphate buffer (20 mM, pH 7.5) / DMSO (v/v, 7:3). After the gel was removed by filtration, the reduced peptide solution was mixed with **3** (6.4 μmol; from 100 mM stock solution in DMSO). The mixture was diluted to 1.0 mL in phosphate buffer (20 mM, pH 7.5) / DMSO (v/v, 3:7). It was reacted for 7 hours at 45 °C in the dark with vigorous shaking, acidified with aqueous formic acid, and purified with reverse-phase high performance liquid chromatography (LC-20AD, Shimadzu) equipped with a XTerra Prep MS C18 column (10 x 50 mm, Waters). The resulting caged binder was separated using a 15–100% linear gradient of MeOH containing 0.1% formic acid during 15 min at a flow rate of 4 mL per minute. It was lyophilized completely and characterized by LC-MS and LC-MS/MS (Figure S6). The overall yield was 6.6% (0.21 mg, 0.19 μmol).

2.4. Molecular docking simulation of caged binder to GST using sievgene of myPresto

For the docking simulation, the structure of the caged binder (i.e., NTVSC*HGF) was created by ChemDraw Ultra (version 11.0) and converted to a mol file. Docking of the ligand to *S. japonicum* GST (PDB: 1UA5) was performed with MolDesk Basic (version 1.1.45, Imsbio Inc., Japan) under a graphical-user interface (GUI) of several myPresto programs [10] as follows. First, the mol file was further converted to a mol2 file. Second, the target GST protein was stripped out of the co-crystalized glutathione, and converted to a pdb file format. Finally, the ligand and the glutathione-subtracted GST input files were entered and docked using sievgene program [11] of myPresto (version 5.000). For the docking, thirty separate poses were taken and their free energies were calculated. Nine poses out of the top 10 docking models, including the best one (Figure 4 in the main text), resembled to each other; the azido group of the caged binder and the 69th methionine of GST were located very close to each other (data not shown). The binding geometry of the best docking model was supported experimentally by an exclusive covalent conjugation between the caged binder and GST, which was

proved by MS/MS analysis of the trypsinized fragment of the conjugated GST (also see Figure 4 in the main text and Figure S7).

Note: Whole amino-acid sequence of *S. japonicum* GST is: MSPILGYWKI KGLVQPTRLL LEYLEEKYEE HLYERDEGDK WRNKKFELGL EFPNLPYYID GDVKLTQSMA IIRYIADKHN MLGGCPKERA EISMLEGAVL DIRYGVSRIA YSKDFETLKV DFLSKLPEML KMFEDRLCHK TYLNGDHVTH PDFMLYDALD VVLYMDPMCL DAFPKLVCFK KRIEAIPQID KYLKSSKYIA WPLQGWQATF GGGDHPPK

(The conjugated methionine at the 69th positon is colored in red, and the corresponding trypsinized fragment is underlined.)

References

1. Suchanek, M.; Radzikowska, A.; Thiele, C. Photo-leucine and photo-methionine allow identification of protein-protein interactions in living cells. *Nat. Methods* **2005**, *2*, 261–267, doi:10.1038/nmeth752.
2. Masuda, Y.; Aoyama, K.; Yoshida, M.; Kobayashi, K.; Ohshiro, T.; Tomoda, H.; Doi, T. Design, Synthesis, and Biological Evaluation of Beauveriolide Analogues Bearing Photoreactive Amino Acids. *Chem. Pharm. Bull (Tokyo)* **2016**, *64*, 754–765, doi:10.1248/cpb.c16-00095.
3. Berg, M.; Michalowski, A.; Palzer, S.; Rupp, S.; Sohn, K. An in vivo photo-cross-linking approach reveals a homodimerization domain of Aha1 in S. cerevisiae. *PLoS ONE* **2014**, *9*, e89436, doi:10.1371/journal.pone.0089436.
4. Wang, L.; Hisano, W.; Murai, Y.; Sakurai, M.; Muto, Y.; Ikemoto, H.; Okamoto, M.; Murotani, T.; Isoda, R.; Kim, D., et al. Distinct metabolites for photoreactive L-phenylalanine derivatives in Klebsiella sp. CK6 isolated from rhizosphere of a wild dipterocarp sapling. *Molecules* **2013**, *18*, 8393–8401, doi:10.3390/molecules18078393.
5. Schwyzer, R.; Caviezel, M. p-Azido-L-phenylalanine: a photo-affinity 'probe' related to tyrosine. *Helv. Chim. Acta.* **1971**, *54*, 1395–1400, doi:10.1002/hlca.19710540521.
6. Ge, S.-S.; Chen, B.; Wu, Y.-Y.; Long, Q.-S.; Zhao, Y.-L.; Wang, P.-Y.; Yang, S. Current advances of carbene-mediated photoaffinity labeling in medicinal chemistry. *RSC Advances* **2018**, *8*, 29428–29454, doi:10.1039/C8RA03538E.
7. Murale, D.P.; Hong, S.C.; Haque, M.M.; Lee, J.S. Photo-affinity labeling (PAL) in chemical proteomics: a handy tool to investigate protein-protein interactions (PPIs). *Proteome. Sci.* **2016**, *15*, 14, doi:10.1186/s12953-017-0123-3.
8. Brase, S.; Gil, C.; Knepper, K.; Zimmermann, V. Organic azides: an exploding diversity of a unique class of compounds. *Angew. Chem. Int. Ed. Engl.* **2005**, *44*, 5188–5240, doi:10.1002/anie.200400657.
9. Taki, M.; Inoue, H.; Mochizuki, K.; Yang, J.; Ito, Y. Selection of Color-Changing and Intensity-Increasing Fluorogenic Probe as Protein-Specific Indicator Obtained via the 10BASE(d)-T. *Anal. Chem.* **2016**, *88*, 1096–1099, doi:10.1021/acs.analchem.5b04687.
10. Fukunishi, Y.; Mikami, Y.; Nakamura, H. The filling potential method: A method for estimating the free energy surface for protein-ligand docking. *J. Phys. Chem. B* **2003**, *107*, 13201–13210, doi:10.1021/jp035478e.
11. Fukunishi, Y.; Mikami, Y.; Nakamura, H. Similarities among receptor pockets and among compounds: Analysis and application to in silico ligand screening. *J. Mol. Graph. Model.* **2005**, *24*, 34–45, doi:10.1016/j.jmgm.2005.04.004.

© 2018 by the authors; licensee MDPI, Basel, Switzerland. This article is an open access article distributed under the terms and conditions of the Creative Commons Attribution (CC BY) license (http://creativecommons.org/licenses/by/4.0/).
